# Cross-Platform Implementation of an SSVEP-Based BCI for the Control of a 6-DOF Robotic Arm

**DOI:** 10.3390/s22135000

**Published:** 2022-07-02

**Authors:** Eduardo Quiles, Javier Dadone, Nayibe Chio, Emilio García

**Affiliations:** 1 Instituto de Automática e Informática Industrial, Universitat Politècnica de València, 46022 Valencia, Spain; jadara@etsid.upv.es (J.D.); nachch@posgrado.upv.es (N.C.); egarciam@isa.upv.es (E.G.); 2 Facultad de Ingeniería, Ingeniería Mecatrónica, Universidad Autónoma de Bucaramanga, Bucaramanga 680003, Colombia

**Keywords:** brain computer interface (BCI), Electroencephalography (EEG), Steady-State Visually Evoked Potential (SSVEP), robot control, C++

## Abstract

Robotics has been successfully applied in the design of collaborative robots for assistance to people with motor disabilities. However, man-machine interaction is difficult for those who suffer severe motor disabilities. The aim of this study was to test the feasibility of a low-cost robotic arm control system with an EEG-based brain-computer interface (BCI). The BCI system relays on the Steady State Visually Evoked Potentials (SSVEP) paradigm. A cross-platform application was obtained in C++. This C++ platform, together with the open-source software Openvibe was used to control a Stäubli robot arm model TX60. Communication between Openvibe and the robot was carried out through the Virtual Reality Peripheral Network (VRPN) protocol. EEG signals were acquired with the 8-channel Enobio amplifier from Neuroelectrics. For the processing of the EEG signals, Common Spatial Pattern (CSP) filters and a Linear Discriminant Analysis classifier (LDA) were used. Five healthy subjects tried the BCI. This work allowed the communication and integration of a well-known BCI development platform such as Openvibe with the specific control software of a robot arm such as Stäubli TX60 using the VRPN protocol. It can be concluded from this study that it is possible to control the robotic arm with an SSVEP-based BCI with a reduced number of dry electrodes to facilitate the use of the system.

## 1. Introduction

Robotics has been successfully applied to help people with disabilities perform different tasks. Many individuals suffer motor limitations as a consequence of strokes, traumas, muscular dystrophies, cerebral palsies, or various neurodegenerative diseases such as amyotrophic lateral sclerosis (ALS). For these patients, daily activities such as reaching and moving objects can be an important problem. Assistance or collaborative robots have been developed to help subjects with motor limitations in performing daily tasks and to allow them a greater degree of autonomy. However, the handling of these robots through buttons or joysticks remains an obstacle for many users affected by motor dysfunctions.

Brain-computer interfaces (BCIs) are an interesting alternative for robot control by people with severe motor limitations. BCIs are non-muscular communication and control systems that a person can use to communicate his intention and act on the environment from measurements of brain activity [1,2,3,4,5,6,7,8]. The term was introduced by Jacques Vidal in 1973 [9] and in 1999 the definition of a BCI system was formalized during the first international meeting on BCI technology [8].

BCIs include sensors that record brain activity and software that processes this information in order to interact with the environment by means of actuators. In the majority of implementations, non-invasive BCIs based on the acquisition of EEG signals are used [10].

BCI’s potential as an assistive technology system was the main driver for its development. Patients with limited communication and movement capabilities can benefit from this technology, which includes communication protocols such as spellers [11,12,13,14] control of robot arms and neuro-prosthesis [15,16,17,18,19], control of motorized wheelchairs [20,21,22,23], home automation systems [24,25], virtual reality [26] and patients with paralysis that they can restore interaction with their environment [8].

Different paradigms have been used in BCI applications based on EEG for robot control, such as Motor Imagery (MI) [19,27,28,29,30,31,32,33], P300 evoked potentials [14,34,35], or Steady-State Visually Evoked Potentials (SSVEP) [36,37,38,39,40,41,42,43,44,45,46,47,48].

In [16] a rehabilitation application based on the control of a robot arm is shown to perform tasks of grasping parts by using a MI paradigm. The application allows two-dimensional control of a robot arm. The imagined movement of left, right, and both hands and relaxation allow movement to the left, right, up, and down of the robot [17].

With the SSVEP paradigm, different studies have been performed to control a robotic arm or hand, seeking that people with muscular or neuromuscular disorders can interact and communicate with their environment [47,49]. The tasks performed with a robotic hand have been opening or closing the hand [50] or giving the order for the robot hand to make a gesture (greeting, approval, or disapproval) [51].

Combinations of two or more paradigms have also been proposed to control a robotic arm such as P300/SSVEP [52,53], SSVEP/mVEP [54], SSVEP/MI/Electromyography (EMG) [55], SSVEP/Facial gestures [56], control of a robotic arm and a wheelchair by SSVEP/cervical movements [57], SSVEP/EOG [58], SSVEP/Eye [59], SSVEP/Computer Vision [60], SSVEP/MI [55], and SSVEP/P300/MI [61].

Existing studies of the application of BCI systems to the control of assistive devices are limited to pilot tests, and their use outside the laboratory has not been generalized [49,62,63]. Some of the drawbacks that prevent the widespread use of BCIs in patients are the high cost of hardware and development of the BCI [62], the laborious preparation of the interface (i.e., placement of the electrodes to acquire the EEG signal) [10], the need for training on the part of the subject in the use of the BCI and its calibration [64].

The use of open-source software (OSS) has made it possible to implement low-cost BCI applications without having to pay for user licenses. There are different types of OSS that can be implemented in projects related to BCIs [65,66]. These software platforms receive brain signals and allow scenarios to be designed that interact with external or simulation devices. Likewise, these platforms allow the processing of electroencephalographic signals such as filtering, feature extraction, and classification [67]. Some examples of commercial and free software platforms with which interaction between users and devices can be carried out for the implementation of a BCI are Matlab [68,69,70], Labview [71,72], Openvibe [73,74,75], BCI2000 [76,77,78], BCI++ [79], o OpenBCI [80]. Tools for offline analysis of EEG signals have also been developed, such as those developed in Matlab by the Swartz Center of Computational Neuroscience (SCCN) [81].

In this study, Openvibe is used to acquire, filter, process, classify and visualize EEG signals in the development of the BCI application. Scenario design is performed with toolboxes and can be used in real-time. Openvibe is a platform developed in C++. It allows working under Linux or Windows operating system. It is licensed under the Affero General Public License (AGPL), which is a copyleft license derived from the GNU General Public License designed for cooperation within the research community and was made by the Institut National de Recherche en Informatique et en Automatique (INRIA) [73].

Openvibe has been used for multiple applications and with different types of paradigms such as motor imagination to control a robotic arm or a robotic hand [30,31], motor imagination in neural plasticity with a wrist exoskeleton [32], with the P300 paradigm for the control of an electric chair [23], P300 for the control of a manipulator robot [35], surface electromyographic signals for the control of a functional electrical stimulator (FES) [82], neurorehabilitation [28,33,83], music [84], mobile robots [85] or processing with motor imagination or P300 [86,87,88,89] with different amplifiers [90].

Different types of interfaces or amplifiers have been used to obtain electroencephalographic signals through the Openvibe platform, such as Neurosky under the motor imagination paradigm [29] or Emotiv with the P300 paradigm [90]. An Enobio amplifier from Neuroelectrics is used in this study. Enobio has been used for the acquisition and processing of EEG signals in research related to different BCI applications, such as subjective behaviors in marketing [91] and other activities [92,93,94,95,96].

This work implements and tests a BCI application based on scalp EEG for the control of a robot arm with minimum requirements at the hardware and software levels from the point of view of the programmer and the user. These design requirements are addressed with the use of open-source BCI software. A cross-platform application is developed to interface OpenVibe and the proprietary software that controls the Staübly robot arm.

With these design specifications, the SSVEP paradigm has been selected, which, compared to the other paradigms commonly used in EEG BCIs, provides greater communication speed [7,10,97,98], the subject requires less training time [10,55,97,98,99], and can be operated with fewer electrodes placed on the occipital region [46,48,50,51,100,101,102,103]. The SSVEP signal appears in the visual cortex when the subject observes intermittent stimuli [104], and its response depends on the subject’s attention [105,106,107] and the size, shape, and frequency of the stimulus [7,19,108].

Minimum requirements are also considered for the BCI experimental subject to facilitate the use of the control system. In order to simplify the acquisition of the EEG signal, dry electrodes are chosen, avoiding the use of electroconductive gel. To also improve the readiness of implementation, just eight electrodes are selected.

The SSVEP-based BCI system designed has been tested with five healthy subjects without previous experience in BCIs. The results suggest that the control of the robot arm through the integration of an open BCI software platform with the software program developed to control the robot is feasible. The proposed system allows controlling the robot arm with a level of demand acceptable to the user.

The prototype developed is presented in the following sections. EEG signal processing and communication between the robot arm and the BCI application are described. The performance of the participants with the BCI is analyzed and compared with previous studies in the area. Finally, the results are discussed, and the conclusions of the study are presented.

## 2. Materials and Methods

### 2.1. System Description

**Figure 1:**
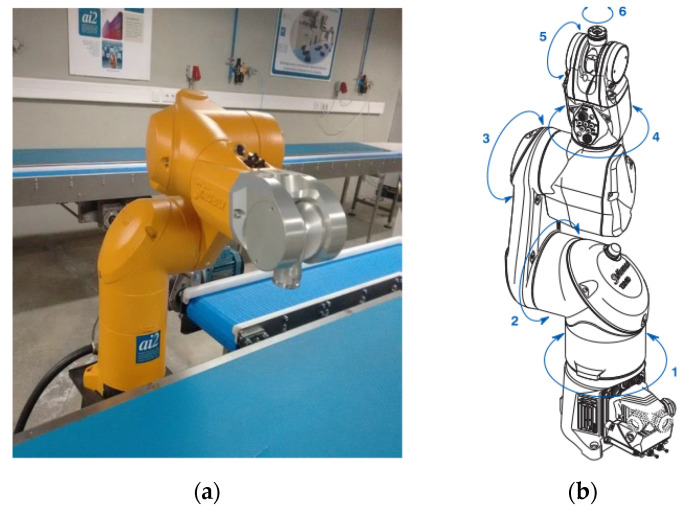
Robot Stäubli TX60. (**a**) robot arm in the lab; (**b**) degrees of freedom scheme.

The SSVEP BCI control system proposed in this work is composed of two main subsystems: The SSVEP processing system and the robot system. EEG signals are wirelessly transmitted from the EEG Enobio amplifier. The EEG signal is then processed to obtain the control signal for the robotic arm. The SSVEP BCI communicates with the robotic arm via a TCP/IP communications protocol. The robotic arm is a six-axis industrial manipulator model TX60 from Stäubli [109]. It is a light-duty robot arm with a maximum load of 9 kg in certain positions. The robot arm weighs 3.5 kg, and its maximum reach is 670 mm (Figure 1).

As previously stated, among the different software platforms available for the acquisition, processing, and classification of EEG signals [76,110,111,112], Openvibe has been selected [97,98,113]. This software was developed by INRIA [113] in order to design, test, and use brain-computer interfaces. Its programming is based on block diagrams and allows the EEG signals to be acquired, filtered, conditioned, classified, and visualized. Openvibe is compiled in C++, so it allows quick and easy integration of the communication library with the robot.

**Figure 2:**
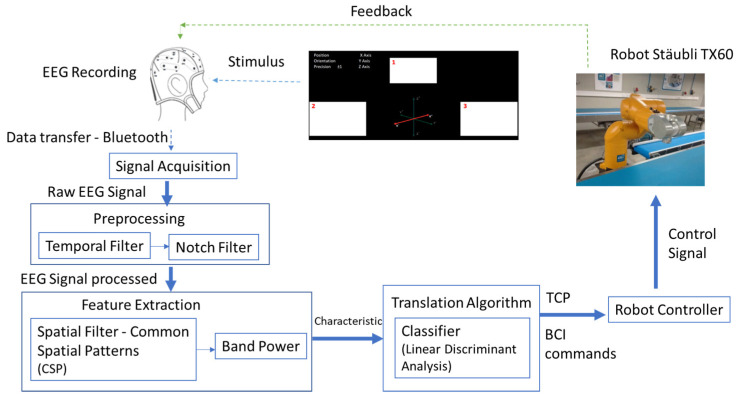
SSVEP-BCI methodology for robotic arm control.

Figure 2 shows the architecture of the SSVEP BCI control system. The different modules are explained in the following sections.

### 2.2. Stimulus Generation

Stimulus generation for the elicitation of the SSVEP can be based on light-emitting diodes LEDs [114] or monitors [46,51,101,103,108,115]. In this study, intermittent visual stimuli are presented on the computer screen.

**Figure 3:**
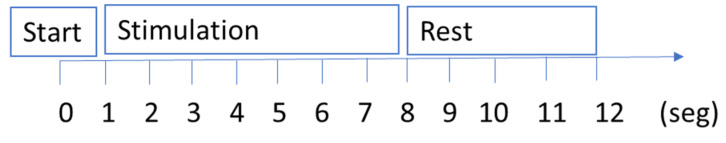
Timing of a single SSVEP trial.

The stimulus type is a white square with a black background [115,116,117]. The size of the stimulus and the location of the stimuli on the screen is configured in Openvibe. Frequencies used are 12, 15, and 20 Hz [114]. These frequencies are multiples (1/5, 1/4, 1/3) of the update rate from a 60 Hz LCD screen (Figure 3).

The experimental procedure to train the BCI spatial filters and classifier has 32 trials arranged in 8 runs. Every run has four trials. Every trial has a length of 12 s and consists of three sections: (1) stimulus presentation (arrow positioning), (2) visualization period, and (3) rest period and stimulus change.

**Figure 4:**
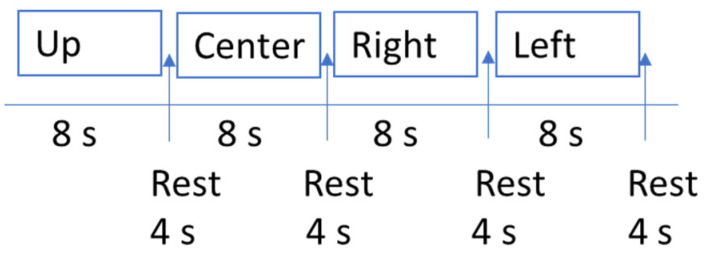
Time duration for starting on stimulation frequency and resting period in one run.

The duration of the stimulus is set to 7 s, the duration between stimuli or interval time of trials is set to 4 s, and the delay of the stimulus is 1 s (Figure 3). The stimuli are shown in sequence, and each stimulus is repeated eight times (Figure 4).

**Figure 5:**
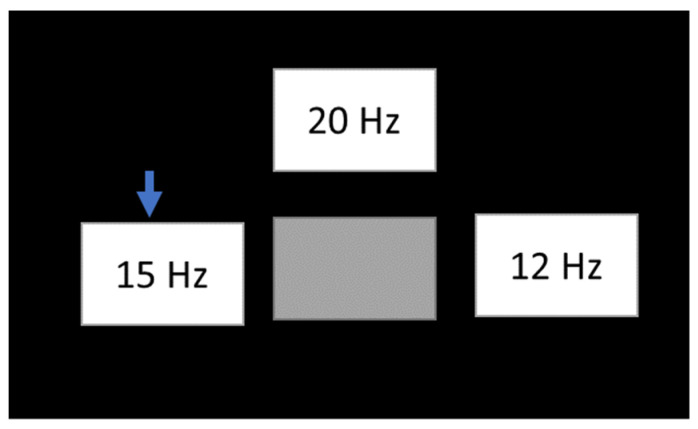
Stimuli frequencies.

Subjects observed on an LCD screen the three stimuli placed on the top, right, and left parts of the computer screen. The center square has the same color as the black background. The execution begins with the positioning of the arrow in one of the four stimuli, and then the stimuli begin to oscillate at 20 Hz (upper box), 15 Hz (left box), and 12 Hz (right box). Simultaneously, the subject must focus on the stimulus for 7 s. Then, the stimuli stop flashing, and the arrow is repositioned on one of the other stimuli. The sequence is random in each run (Figure 5).

### 2.3. Signal Acquisition

Several factors have been taken into account when selecting hardware and software components for the EEG-based BCI. Given the interest in a compact and portable solution for BCI control, Enobio digital amplifier from Neuroelectrics [118] was selected to acquire the EEG signals. The Enobio amplifier was developed for BCI research. It was chosen for its wireless technology and dry electrodes that facilitate the experimental setup.

**Figure 6:**
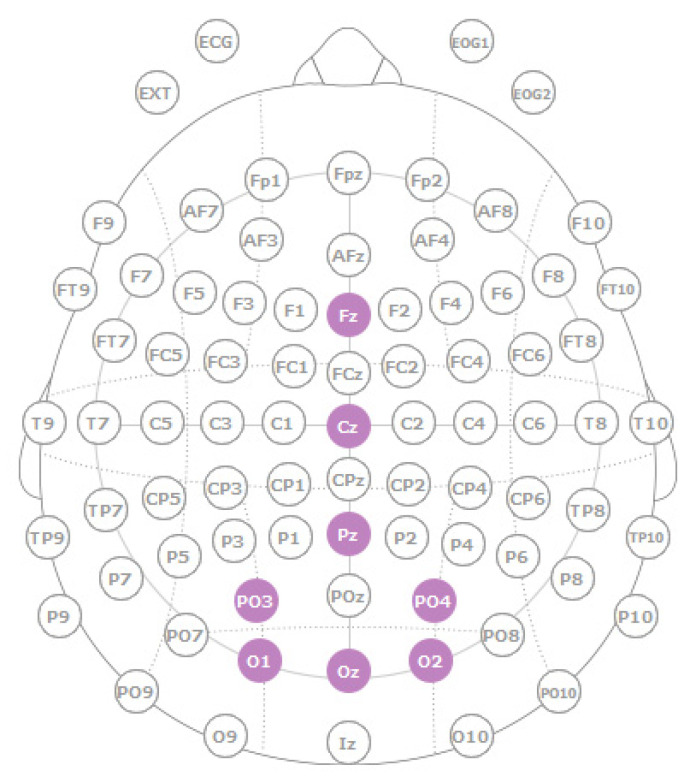
Electrode disposition according to the international system 10–20.

The EEG signal was acquired through channels O1, O2, Oz, PO3, PO4, Pz, Cz, and Fz around the occipital area according to the standard 10–20 electrode location system (Figure 6). Ground and reference electrodes were placed in the subject’s earlobe. The EEG signal was recorded using a sampling rate of 500 Hz and band-pass filtered between 2 and 100 Hz with an activated notch filter at 50 Hz. The sampled and amplified EEG signal is then sent to the computer via Bluetooth.

### 2.4. Signal Processing

**Figure 7:**
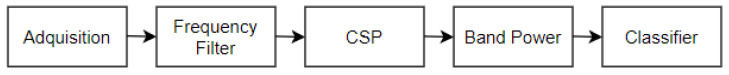
Signal Processing Procedure.

The EEG signal is sent from Enobio to Openvibe through the Openvibe Acquisition Server module [113]. The EEG signal is processed in a five-step process: preprocessing, feature extraction, classification, command translation, and feedback to the BCI user. Its programming is based on block diagrams and allows the signals to be acquired, filtered, conditioned, classified, and visualized (Figure 7).

Considering that the cognitive activity of interest in this study is in the range of 0.2–40 Hz, a fourth-order Butterworth band-pass filter between 6–40 Hz was applied to the EEG signal. According to [119], the SSVEP paradigm is less sensible to artifacts, due to its high signal-to-noise ratio (SNR) and robustness, than other typical BCI paradigms [120] said that SSVEP are little affected by muscular artifacts such as blinking and facial muscles’ EMG. As one of the objectives of this work was to research into practical applications of BCI systems in non-clinical settings, SSVEP was selected because of its high signal-to-noise ratio (SNR). Nevertheless, appropriate processing and filtering of artifacts must be conducted in every BCI system.

For feature extraction, we use a spatial approach [121]. A common spatial patterns (CSP) filter selects the best characteristics from the EEG signal. The CSP algorithm produces spatial filters that maximize the variance of bandpass-filtered EEG signals from one class while minimizing their variance for the other class [122,123,124,125,126].

The power spectrum was extracted in the considered frequency bands, respectively 19.75–20.25 Hz for 20 Hz flashing frequency, 14.75–15.25 Hz for 15 Hz flashing frequency and 11.75–12.25 Hz for 12 Hz flashing frequency. For single-trial data (7 s length), a 0.1-s sliding window was applied to extract the signal features. The window length was 0.5 s. A logarithmic mapping is applied to the power average, as it assists in the improvement of the classification performance [127].

In order to classify the features extracted, a linear discriminant analysis (LDA) classifier was used. The aim of LDA is to adjust a hyperplane that can separate the data representing the different classes [67,128]. This classifier is popular and efficient for BCI. For each condition, the training set was used to select the features and to train the LDA classifier on these features. Then, the trained LDA classifier was used to classify the features extracted from the test set [129,130,131].

Cross-validation was used in this study to validate the LDA classifier. The idea was to repeatedly divide the set of trials in a BCI timeline into two non-overlapping sets, one used for training and the other for testing. Cross-validation is typically used in Openvibe in a range from 4 to 10 partitions. In this case a 10-fold cross-validation method was used. The LDA classifier was trained on 90% of the feature vectors and tested on 10%, 10 times.

### 2.5. GUI for Robotic Arm Control

**Figure 8:**
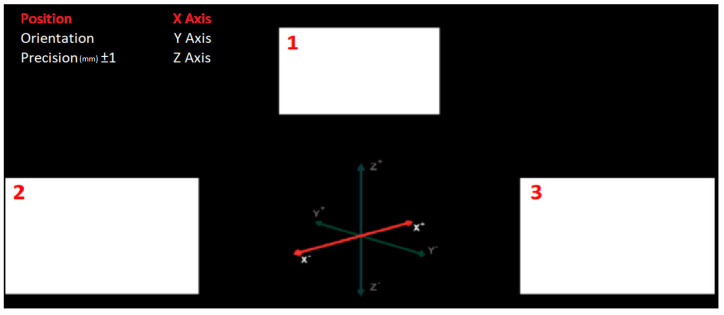
GUI for the control of the robotic arm.

To control the robotic arm, six degrees of freedom are available in position (linear control) and orientation (angular control). The graphical user interface (GUI) designed for the control of the robot arm is shown in Figure 8. Both in the upper left corner of the application screen and on the coordinate axes in the center, the user can visualize the active degree of freedom to control.

The GUI shows the three visual stimuli used to elicit the SSVEP. Stimulus 1 allows changing the degree of freedom. It is oscillating at 20 Hz, and when the subject selects this stimulus, in case of having the position selected, it will alternate between the three main axes (X, Y, and Z) while, in case of having the orientation selected, it would alternate between the three main angles (alpha, beta, and gamma). Stimulus 2, whose frequency is 15 Hz, increases the position and angle negatively, while stimulus 3, programmed at a frequency of 12 Hz, increases them positively.

The increases are related to the selected precision and the degree of freedom to control, this magnitude being millimeters in the case of linear movement or degrees in the case of angular movement. The subject can vary the millimeters or degrees of movement of the robot with the precision indicator located in the upper left corner of the GUI.

### 2.6. Robot Communication Module

**Figure 9:**
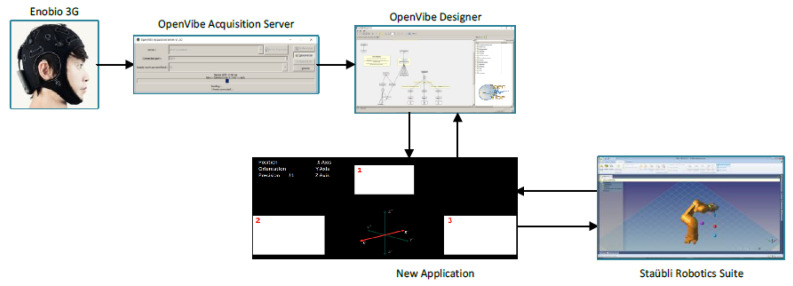
Control signal flowchart.

Figure 9 shows the block diagram for the robot arm control. Openvibe Acquisition Server acquires the EEG signal from Enobio. Openvibe Designer is used for the treatment of the received signal and the classification of the subject’s intention. Through the virtual reality peripherals network (VRPN), the application designed in Openvibe is connected with an external application in Visual Studio. The VRPN protocol has two servers, Analog VRPN Server and Button VRPN Server. The analog server is capable of receiving a connection from an analog client and sending analog signals. The Button VRPN Server is simply a digital server that, receiving the connection from a digital client, can send logical signals similar to the mechanism of a button. In the study, digital servers (Button VRPN Server) have been used to carry out actions (Table 1). The protocol used is TCP/IP, which guarantees the order and reception of the data sent, and also allows communication to start and end in a controlled manner.

In Visual Studio, a program has been developed that receives the output of the classifier in Openvibe Designer and elaborates the control action to act on the robot through a series of events. This program sends the robot the modification of the position or orientation according to the precision and wishes of the subject.

**Figure 10:**
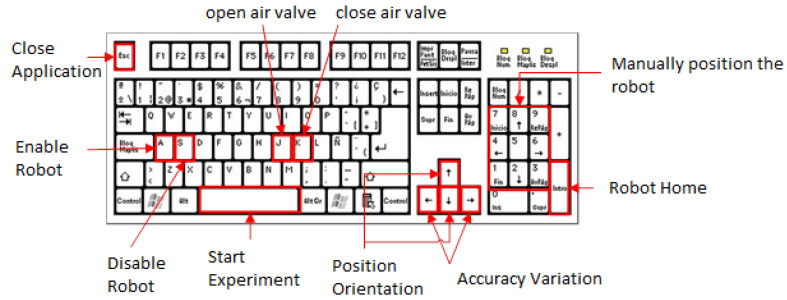
Combination of softkeys and their functionality.

In addition, keyboard-configured security controls have been implemented that allow the experimenter to directly control the execution of the program. Figure 10 summarizes the selected set of keys and their use. The application is launched with the Space key. Once started, the robot is disabled for safety. The programmer can enable or disable the robot’s movements using the appropriate keys. It should be noted that disabling the robot does not cut communication, so it is a useful tool for debugging the response obtained.

The J and K keys allow the opening and closing of a small pneumatic solenoid valve. Thanks to this external drive, the robot can be equipped with a claw to carry out pick and place or similar tasks. With the arrows, it is possible to navigate through the menu. The user can select whether to control the position or orientation of the robot, as well as the precision (millimeters or degrees of freedom). Nine final positions have been programmed for the robot so that they maintain the same height level and vary their position on the plane. In this way, objects can be reached with greater speed in the programmed tasks. Finally, the Enter key returns the robot to its initial position.

**Figure 11:**
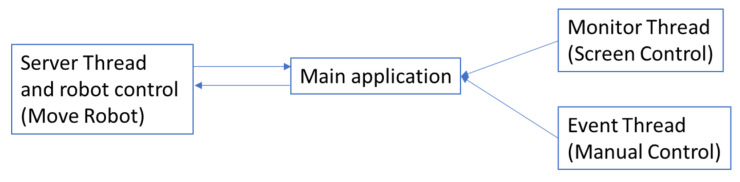
Program execution threads.

Concurrent programming has been used to guarantee the correct operation of the program. The robot needs to be constantly communicating, so it must always have an active server. Furthermore, the application must guarantee that the stimuli maintain the same blinking frequency to avoid false positives and even erroneous control actions. Finally, the screen is continually refreshing itself, and there are a number of external events coming from the keyboard that must be attended to, and actions are taken accordingly. For this reason, the main application runs sequentially and has three execution threads (Figure 11):

- Server thread, in charge of making, maintaining, and recovering the connection with the robot.

- Monitor thread, in charge of refreshing the screen every time there is a modification in it.

- Event thread, responsible for managing all events external to the application and coming from the keyboard.

### 2.7. Subjects

A total of 5 healthy volunteers (3 males and two females; aged 19–30 years) with normal or corrected to normal vision participated in this study. The participants were students from the Universitat Politècnica de València. None of them had previous experience with BCIs. A medical history of epilepsy or the intake of psychoactive drugs were exclusion criteria for this experiment, and none of the participants was rejected for these causes.

Informed consent was obtained from all individual participants included in the study. Subjects were informed about the experimental procedure. Subjects were sitting in front of an LCD screen. They were instructed to focus their attention on the stimulus indicated on the computer screen. They were also instructed to avoid muscle and eye movements and to have a comfortable and relaxed position throughout the experiment.

### 2.8. Experimental Procedure

**Figure 12:**
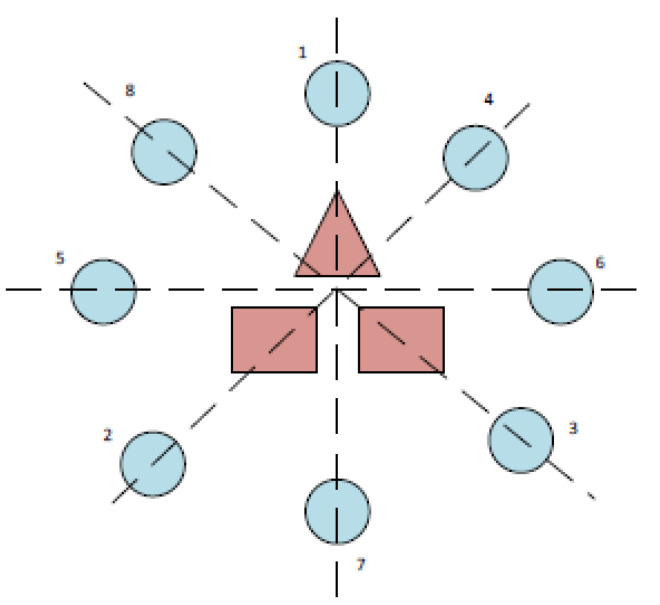
Position of the targets and order of appearance.

**Figure 13:**
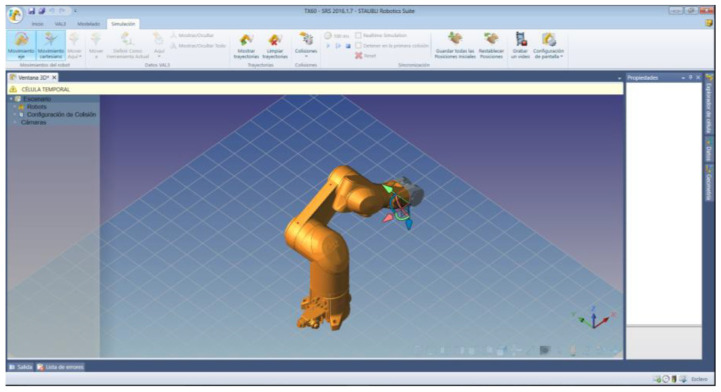
Stäubli Robotic Suite environment showing the rotation of the extreme joint of the robot.

The experimental procedure has followed the recommendations established in [132]. The experimental subjects have to guide the robot arm to eight positions in a 360° range according to the sequence of numbers shown in Figure 12. The rotation of the extreme joint of the robot has been controlled, corresponding to the degree of freedom 6 of Figure 1b. The movement is visualized in the Stäubli simulation environment (Figure 13) and carried out by the 6th articulation of the robot arm. The subjects must rotate this robot arm articulation to the angular positions indicated by a series of circular targets that appear sequentially. To do this, the 12 Hz visual stimulus allows for clockwise rotation and the 15 Hz visual stimulus for counter-clockwise rotation. Once the required rotation is reached, the target is confirmed with the activation of the triangular stimulus that oscillates with a frequency of 20 Hz.

**Figure 14:**
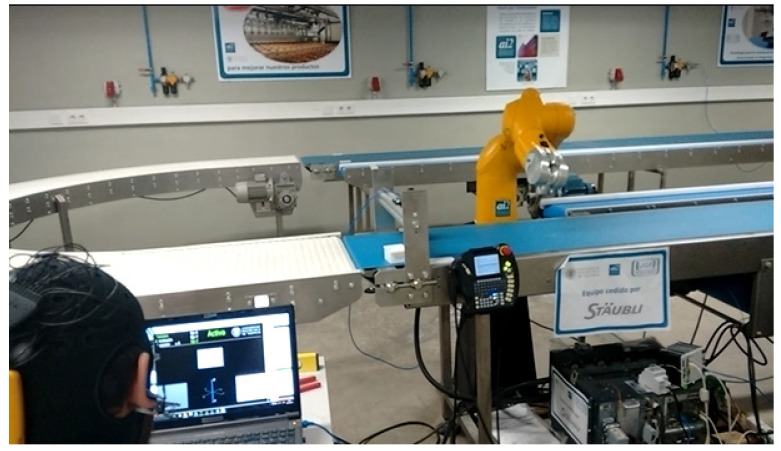
SSVEP BCI **c**ontrol of the Stäubli robotic arm.

Given that targets are distributed every 45°, Table 2 reflects the optimal theoretical movement sequence of the task. The triangular stimulus is initially aimed at the first target, so no turn is necessary. In order to achieve the second objective, a counter-clockwise movement of 135° is required, which corresponds to 45 theoretical steps in the program. In order to carry out the test, a minimum of 285 turning movements are required without counting the shots. The experimental setup is shown in Figure 14.

## 3. Results

Table 3 shows the average time it takes each subject to complete the task. The average has been made considering the number of trials carried out by each subject. It is observed how subjects C, D, and E finished every experimental run in just over three minutes. These subjects are capable of selecting about three targets per minute with the complexity required by reaching and confirming every requested rotation (combined actions). Subjects A and B did not achieve good control of the BCI system and did not complete the full number of trials in the experiment.

**Figure 15:**
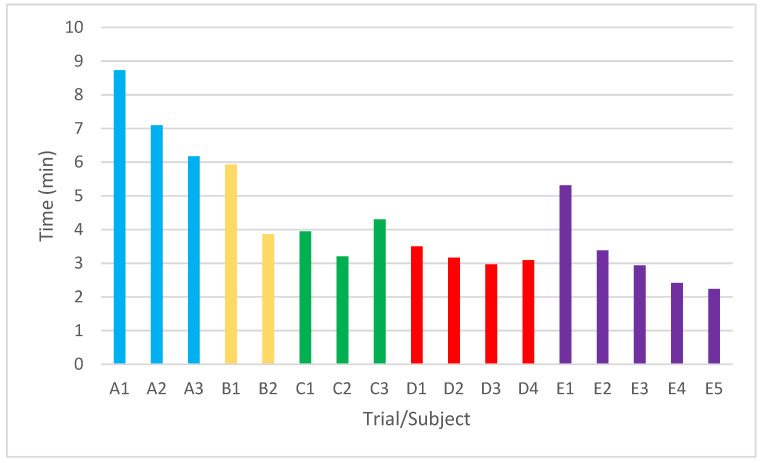
Evolution of the time to complete the task in each attempt of the five subjects.

Figure 15 shows the evolution of learning for each subject, the time spent in each attempt, and the attempts made by each one. It is concluded that subjects A, B, D, and E had a continuous improvement in the task from their first attempt to the last attempt. In subject A the trial time decreased by 29.28%, in subject B by 34.84%, in subject D by 11.64%, and subject E reduced the time by 57.80%. Subject C decreased his time from the first to the second attempt, but in the last attempt, it increased by 8.89%, possibly due to the accumulated fatigue from task repetitions.

**Figure 16:**
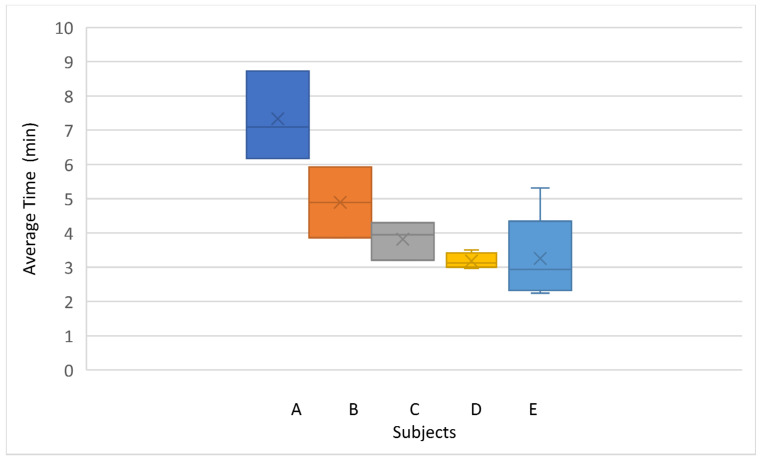
Average total time to complete task per subject.

Figure 16 shows the average time of the attempts made by each subject. The best average time was obtained by subject D with four attempts and a total time of 3.18 min.

Table 4 compares the average success rate of the subjects after performing the experimental tasks according to the minimum number of movements required shown in Table 2. The success rate has been evaluated according to Equation (1). The average, as in the previous case, has been calculated based on the number of trials carried out.

Trial Success (%) = (theoretical movement/experimental movement) × 100(1)

**Figure 17:**
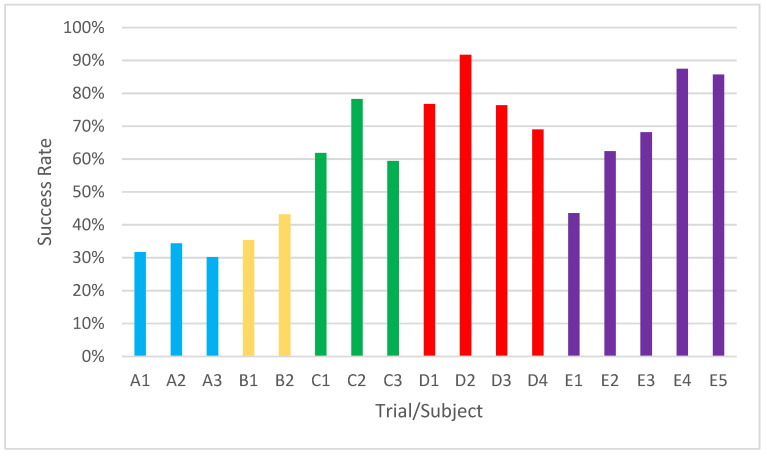
Percentage of success of each subject for each attempt.

Figure 17 shows the percentage of movements calculated with respect to the theoretical minimum number of movements. Subjects A and B did not have a good performance. Their percentage for each attempt was below 50%. Subjects C, D, and E had performances above this value, with subject C achieving a peak performance in one of his attempts of 78.3%, subject D of 91.68% and subject E of 87.5%.

**Figure 18:**
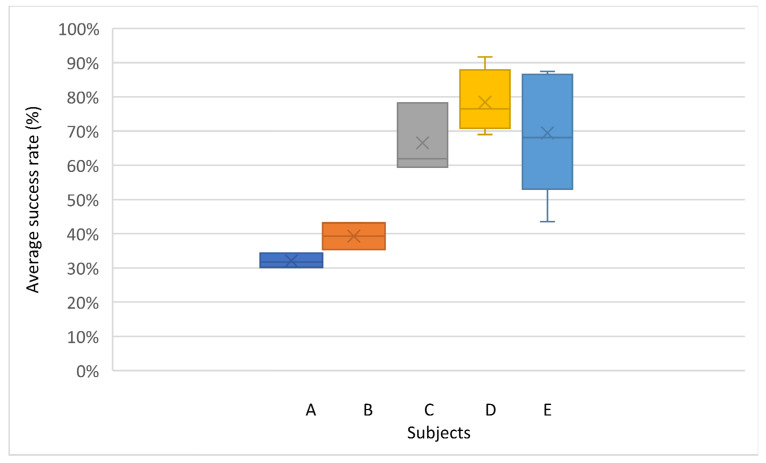
Distribution and average percentage of success of each subject.

Figure 18 shows that the average success percentage was less than 40% in subjects A and B, in the range of 60 and 70% for subjects C and E, and above 70% for subject D.

**Figure 19:**
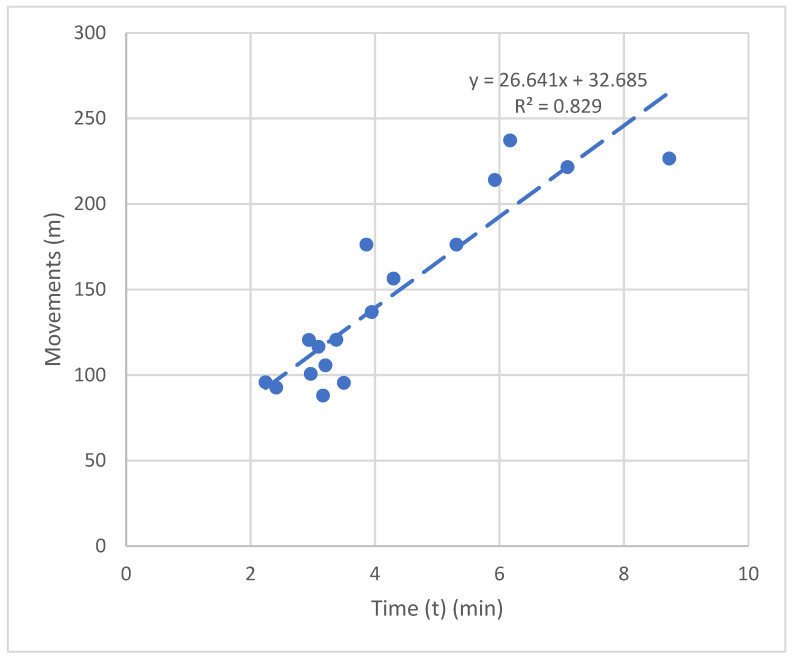
Relationship between time and the number of total movements completed.

In Figure 19, the number of movements performed is displayed as a function of the time spent in completing the task.

The information transfer rate (ITR) was used to evaluate the performance of the BCI system [8,133]. The calculation was made according to Equation (2), where *T* is equal to the stimulus time (7 s) plus the gaze change time (4 s); *N* is the number of stimuli, which in this case was three, and *P* is the precision of the classification (Table 4).

(2) $$\mathrm{ITR}=\frac{60}{T}\;\left[\log_{2}N+{\mathrm{Plog}}_{2}P+\left(1-P\right)\log_{2}\frac{1-P}{N-1}\right]$$

**Figure 20:**
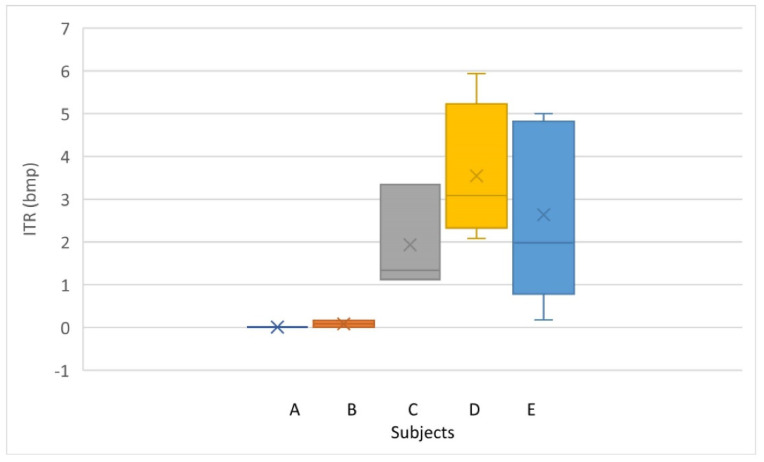
Distribution and average ITR of each subject.

Table 5 and Figure 20 show the ITR of each subject in each trial, as well as the mean value achieved. The best subject had a classification of 91.68% (Table 4) with an ITR of 5.94 bit/min.

## 4. Discussion

The influence of the spatial filtering stage of the EEG signal with respect to the precision of the classifier has been analyzed. For this, three Laplacian filters [134,135] with the weights indicated in Table 6 have been compared with two CSP filters of two and eight dimensions. The CSP algorithm results in spatial filters that maximize the variance of the EEG signals corresponding to one class and minimize it for the other class [122,136].

Table 7 shows the comparison of the spatial filters applied in an LDA classifier. The value obtained is the precision value of the classifier in each subject. From the comparison of the precision measure of the classifier, it can be observed that a significant improvement is obtained with the CSP filters compared to the Laplacian ones. The best response was obtained with a CSP filter of dimension 8, although the difference with respect to the CSP filter of two dimensions is not very significant.

The study carried out is compared with other similar studies below. Out of the seventeen reviewed references, this study is the only one that worked with the Enobio interface with eight dry electrodes. Different studies in robotic application obtained acquisition signals from the occipital/parietal brain areas. There are many interfaces used for acquisition signal such as Enobio (Neuroelectrics) with 32 electrodes [40], Epoc Headset (Emotiv) [51,101,137], Ultracortex (OpenBCI) [45,47], Biosemi [103], Neuracle [46,48], BioRadio (Great Lakes NeuroTechnologies) [39], DSI (Wearable sensing) [42], Mindo 4S (National Chiao Tung University Brain Research Center) [43,138], EEG amplifier Nuamps Express (Neuroscan) [44] and NeuSen W8 (Neuracle) [41]. See Table 8 for a summary.

The references reviewed used a combination of frequencies in the low and medium-range [46,47,48,50,103,137] or in the low range [51,101], while the present study used frequencies in the medium range. This study used a black/white stimulus color as in [51], but with different frequencies, while other studies used stimulus color such as red, blue, and purple, or they used other types of stimuli such as images or names. See Table 9 for a summary.

This study presents a preprocessing and feature extraction process similar to [51]. Both studies used Butterworth fourth-order filters, Common Spatial Pattern (CSP) filters and Band Power (BP) extraction. See Table 10 for a summary.

This study used the Linear discriminant analysis (LDA) as classification, while other studies used other types of classification such as Support vector machine (SVM) [39,42] or Canonical correlation analysis (CCA) [43,45]. Although other classifiers had an accuracy of 85.56% to 95.5%, the LDA classifier had good results for three of the five subjects in this study. Taking into account the task objectives, the classification accuracy can be considered acceptable above 80%. The three subjects that were able to control the BCI had 86% peak average accuracy in a range between 78.30% and 91.68%.

In subsequent studies, the authors intend to improve the classifier training method by coding a wrapper solution as proposed in [139]. This method consists in explicitly defining for each fold which time segments of the signal should belong to the train set and which to the test set, and using this same segmentation for training all of the supervised learning components in all the signal processing scenarios. It is also intended in future studies to compare different classifiers.

Trials have been carried out in subjects without prior experience in BCI applications, so with perseverance and carrying out more trials better results could be achieved. This learning process can be observed in Figure 15 for every participant in the study.

The performance of the participants in the study can be affected by some variables of the study, such as stimulus type and color, the use of LEDs instead of a computer screen for presenting the intermittent stimulus, or selecting another combination of electrodes in the occipital cortex. The authors evaluated the effect of some of these parameters in a previous study [114]. In that work, it was concluded that both white and red colors could be used for medium frequency intermittent stimulus, and both green and red for low frequency, while at high frequency, there are no differences between colors. That is the reason why white stimuli are used in this study.

The perception of the subjects regarding the experiment was that completing the task was feasible, although it required concentration. Participants agreed that the application carries out their wishes except on rare occasions, providing a sense of control. Regarding the placement of the dry electrodes and the perceived pain, all agree that the placement has been simple, and the pain is bearable.

The main limitations of the present work are related to the use of dry electrodes and the number of participants. The use of wet electrodes can improve the quality of the EEG signals obtained and, consequently, the success rate of the experimental trials. However, the aim of this study was to build a BCI system that is easy to apply, with few electrodes, and comfortable to use, avoiding the nuisance of applying the electro-conductive gel. The future success of BCI systems applied to, for instance, assistance robotics or videogames depends on this readiness of use, where the user can wear the electrodes without external assistance.

Regarding the subjects, results showed that although a majority learn and improve in the course of the trials. There is a percentage, in this case, 40% of the subjects where the precision rate is less than 50%. This percentage is close to the reported BCI illiteracy rate [140] in which the subjects are unable to use the BCI. In future studies, the authors intend to increase the number of experimental subjects to corroborate the obtained results.

Finally, a matter of importance is to continue investigating with this paradigm, for example incorporating new functionalities, other analyses with EEG signals algorithms, different stimuli, an embedded application, or the replacement of the screen with LEDs.

## 5. Conclusions

The SSVEP paradigm was used to guide an industrial robot arm through the analysis and processing of the EEG data obtained through an Enobio amplifier with dry electrodes. A cross-platform application was obtained in C++. This platform, together with the open-source software Openvibe, can control a Stäubli robot arm model TX60. Additional security controls through the keyboard were implemented.

Five healthy subjects tried the BCI. Two of them were unable to successfully control the BCI device. This proportion agrees with previous results related to BCI illiteracy rates [140,141]. Regarding the subjects that were able to control the BCI, an average of around 71.5% success (peak performance average of 86%) in the application was obtained in subjects C, D, and E, with an average task completion time of around 3 min. This means that these subjects are capable of performing different actions every 23 s and with a reliability of more than 70% with eight dry EEG electrodes.

It has been confirmed that the black and white colors used in the SSVEP paradigm stimulus have shown good results and that these colors are acceptable to use in an experiment such as the one carried out.

The main conclusion of the study is that it is possible to control in real-time the robotic arm with a cross-platform application developed in C++, using openly available tools to develop an SSVEP-based BCI. The BCI operates with a reduced number of dry electrodes to facilitate the use of the system.

## Figures and Tables

**Table 1:** VRPN communication tags.

TAG	Value	Description
OVTK_StimulationId_ExperimentStart	0X00008001	Simulation Start
OVTK_StimulationId_ExperimentStop	0X00008002	Simulation Stop
OVTK_StimulationId_Label_00	0X00008100	Stimulus for axis change
OVTK_StimulationId_Label_01	0X00008101	Stimulus for negative increase
OVTK_StimulationId_Label_02	0X00008102	Stimulus for positive increase

**Table 2:** Minimum number of movements required for the test.

Target	Anti-Clockwise Rotation (Degrees)	Clockwise Rotation (Degrees)	Theoretical Movements
1	0	0	0
2	135	0	45
3	90	0	30
4	90	0	30
5	135	0	45
6	180	0	60
7	0	90	30
8	0	135	45
Total			285

**Table 3:** Comparative results between subjects.

Subject	Trial Time (min)					
	1	2	3	4	5	Average
Subject A	8.73	7.09	6.17	-	-	7.33
Subject B	5.93	3.86	-	-	-	4.89
Subject C	3.949	3.205	4.300	-	-	3.818
Subject D	3.501	3.165	2.967	3.093	-	3.181
Subject E	5.310	3.379	2.939	2.414	2.241	3.257

**Table 4:** Success rate comparison between subjects.

Subject	Trial Success (%)					
	1	2	3	4	5	Average
Subject A	31.69%	34.33%	30.19%	-	-	32.07%
Subject B	35.32%	43.18%	-	-	-	39.25%
Subject C	61.88%	78.30%	59.45%	-	-	66.54%
Subject D	76.73%	91.68%	76.38%	68.98%	-	78.44%
Subject E	43.56%	62.42%	68.14%	87.50%	85.70%	69.46%

**Table 5:** ITR for every subject and trial.

Subject	Trial ITR (bpm)					
	1	2	3	4	5	Average
Subject A	0.005	0.002	0.018			0.008
Subject B	0.007	0.165				0.086
Subject C	1.336	3.345	1.120			1.933
Subject D	3.107	5.937	3.055	2.081		3.545
Subject E	0.178	1.386	1.982	4.999	4.635	2.636

**Table 6:** Laplacian filter weights.

Laplacian Filter ID	O1	O2	OZ	PO3	PO4	PZ
LF1	1	1	1	−1	−1	−1
LF2	1	1	−2	0	0	0
LF3	1	1	1	0	0	0

**Table 7:** Spatial filter comparison.

	Classifier Precision (%)				
Filter	LF1	LF2	LF3	CSP(2)	CSP(8)
Subject A	52.02	49.84	49.92	65.36	65.47
Subject B	41.17	37.38	37.35	60.17	61.28
Subject C	46.57	39.53	34.92	64.4	65.74
Subject D	44.9	40.79	40.79	61.31	62.12
Subject E	43.28	45.29	45.29	67.46	68.32
Average	45.588	42.566	41.654	63.74	64.632

**Table 8:** Robotic application, acquisition device and electrode characteristics.

Study	Application	Amplifier	Electrodes	Electrodes Placement
Present study	Robotic arm	Enobio	8	Cz, O1, O2, PO3, Oz, PO4, Pz, reference and ground electrodes in the right ear lobe
Al-maqtari et al. [50]	Robotic hand	Ag/AgCl electrodes	3	O1, O2, reference electrode in left ear lobe
Çiǧ et al. [137]	Robotic arm	Emotiv Epoc Headset	14	O1, O2
Pelayo et al. [47]	Robotic arm	Ultracortex	8	No information
Meattini et al. [51]	Robotic hand	Emotiv Epoc Headset	14	O1, O2, P7, P8
Bakardjian et al. [103]	Robotic arm	Biosemi	128	12 occipital channels
Chen et al. [48]	Robotic arm	Neuracle	9	PZ, PO5, PO3, POZ, PO4, PO6, O1, OZ, O2, ground between FZ-FPZ
Cáceres et al. [101]	Robotic arm	Emotiv Epoc Headset	14	O1, O2
Chen et al. [46]	Robotic arm	Neuracle	10	P3,PZ,P4,PO3,PO4,T5,T6,O1,OZ,O2, ground between FPZ and FZ, reference electrode CZ
Sandesh et al. [37]	Robotic hand	Ag/AgCl electrodes	No information	No information
Karunasena et al. [39]	Wrist and robotic gripper arm	BioRadio	1	Oz (Cz - FPz = Ground-Reference)
Sharma et al. [40]	Robotic arm	Enobio	32	Oz-Pz-Fp1
Zhang et al. [42]	Robotic arm	DSI	24	P3,P4,Cz,T5,T6,O1,O2
Chen et al. [41]	Robotic arm	NeuSen W8	8	T5, P3,PZ, P4, T6,O1, Oz, O2
Lin et al. [43]	Robotic arm	Mindo 4S	4	O1, O2
Kaseler et al. [44]	Robotic arm	EEG amplifier	9	P3,Pz, P4, PO3, POz, PO4, O1, Oz, O2
Tabbal et al. [45]	Robotic arm	OpenBCI	8	O1, O2, Oz
Chiu et al. [138]	Robotic arm	Mindo 4S	4	O1, O2

**Table 9:** Stimuli characteristics.

Study	Frequencies (Hz)	Screen	Stimuli Color	Subjects	Session/Block (Trials)
Present study	12, 15, 20	LCD monitor	White/Black	5	32
Al-maqtari et al. [50]	8, 13	LED	Red	2	30
Çiǧ et al. [137]	6.66, 7.5, 8.57, 10, 12	No information	No information	11	No information
Pelayo et al. [47]	7, 11, 15	LED	No information	3	30
Meattini et al. [51]	6, 7.5, 10	LCD monitor	White/Black	No information	No information
Bakardjian et al. [103]	Exp1: 5-12 Exp2: 5-5,4-6-6,7-7,5-8,5-10-12	LCD monitor	Videos	8	No information
Chen et al. [48]	From 8 to 15.2 in 0.3 Hz steps	LCD monitor	Blue	4	4 session—25 trials
Cáceres et al. [101]	6-4, 3-5	LCD monitor	Red-Blue-Purple	6	No information
Chen et al. [46]	8–15 Hz in 0.5 Hz steps	LCD monitor	No information	12	No information
Sandesh et al. [37]	21 Hz	LED	No information	5	2 session—5 trials
Karunasena et al. [39]	6.5, 7.5, 8.2, 9.3	LED	White	3	30 s at each stimulus frequency
Sharma et al. [40]	15	Laptop	Square	1	30 s at each fixation targets
Zhang et al. [42]	9, 9.25, 9.5, 9.75, 10.25, 10.5, 10.75, 11, 11.25, 11.5, 11.75, 12	LCD Monitor	Images	20	400 trials
Chen et al. [41]	9, 9.5, 10, 10.5, 11, 11.5, 12, 12.5, 13, 13.5, 14, 14.5	Laptop	White Name	8	5 blocks of 12 trials
Lin et al. [43]	14.4, 16, 18, 20.6, 24	Monitor	Circles—Black and white	15	3 blocks of 5 trials
Kaseler et al. [44]	8, 57, 10, 12, 15 Hz, 7.96 to 14.86 steps 0.46 Hz	LCD Monitor	Square	2	20 trials in each test
Tabbal et al. [45]	7.5, 10, 12	No information	Red/blue	5	4 blocks of 8 trials
Chiu et al. [138]	14.4, 16, 18, 20.6, 24	Monitor	Circles—Black and white	15	3 blocks of 5 trials

**Table 10:** Signal processing characteristics.

Study	Feature Extraction/ Classification	Accuracy
Present study	Band power (BP) Linear discriminant analysis (LDA)	The average precision was 60.9%, in a range between 30.19% and 91.68%. The average of three of the five subjects was 85.83%
Al-maqtari et al. [50]	FFT, Power Density Spectrum (PDS)	No information
Çiǧ et al. [137]	Hilbert transform (HT) and Multi wavelet transform (MWT) Neural Network and cubic-Support vector machine (SVM)	90%
Pelayo et al. [47]	FFT, SNR	85.56%
Meattini et al. [51]	Band power (BP) Support vector machine (SVM)	The accuracy of reading four states was just under 90%, which is acceptable for the application of gesturing.
Bakardjian et al. [103]	Independent Component Analysis (ICA) and phase-locking value (PLV) Linear discriminant analysis (LDA)	No information
Chen et al. [48]	Canonical correlation analysis (CCA)	95.50 ± 3.00%
Cáceres et al. [101]	FFT, Power spectral density (PSD)	91.65 ± 9.13%
Chen et al. [46]	Canonical correlation analysis (CCA)	92.78%
Sandesh et al. [37]	Wavelet LDA	Accuracy 84% and completion time 44.6 seg
Karunasena et al. [39]	FFT Euclidean distance	Accuracy between 29.6% and 61.8%
Sharma et al. [40]	FFT	Accuracy 79%
Zhang et al. [42]	CCA (Canonical Correlation Analysis) Adaptative FBCCA/Bayesian estimation	Accuracy 95.5%
Chen et al. [41]	CCA (Canonical Correlation Analysis)	Accuracy between 76.67% and 98.33%
Lin et al. [43]	FFT, SNRCCA	Acurracy 90%
Kaseler et al. [41,44]	No information	Accuracy between 60% and 100%
Tabbal et al. [45]	Welch power spectral density/FFT/Singular Value Decomposition (SVD) SVM	The accuracy of the three methods is between 50% and 98.75%
Chiu et al. [138]	FFT, SNR CCA	Accuracy 91.35%

## Abbreviations

The following abbreviations are used in this paper:

BCI	Brain-computer interface
CSP	Common spatial patterns filter
EEG	Electroencephalography
EMG	Electromyography
GUI	Graphical user interface
ITR	Information transfer rate
LCD	Liquid crystal display
LDA	Linear discriminant analysis
mVEP	Motion-onset visual evoked potential
MI	Motor imagery
P300	Event-related potential component
SPSS	Statistical Package for the Social Sciences
SSVEP	Steady-State Visual Evoked Potentials
TCP/IP	Transmission Control Protocol/Internet Protocol
UDP	User datagram protocol
VEP	Visual evoked potential
VRPN	Virtual reality peripherals network
